# Genetically defined systemic autoinflammatory diseases in pediatric patients with Behçet’s disease

**DOI:** 10.3389/fimmu.2026.1897406

**Published:** 2026-08-18

**Authors:** Shaoling Zheng, Pui Y Lee, Qing Zhou, Xu Han, Xuechan Huang, Yukai Huang, Jiajun Chen, Chun Zheng, Xiaolin Fang, Jianhua Yu, Aiwu Wang, Xia Pan, Xiaomin Yu, Tianwang Li

**Affiliations:** 1 Department of Rheumatology and Immunology, The Affiliated Guangdong Second Provincial General Hospital of Jinan University, Guangzhou, Guangdong, China; 2 Division of Immunology, Boston Children’s Hospital, Harvard Medical School, Boston, MA, United States; 3 State Key Laboratory of Common Mechanism Research for Major Diseases, Institute of Basic Medical Sciences & School of Basic Medicine, Chinese Academy of Medical Sciences & Peking Union Medical College, Beijing, China; 4 Liangzhu Laboratory, Zhejiang University, Hangzhou, Zhejiang, China; 5 Department of Laboratory Medicine, The Affiliated Guangdong Second Provincial General Hospital of Jinan University, Guangzhou, Guangdong, China; 6 Department of Hematology, The Affiliated Guangdong Second Provincial General Hospital of Jinan University, Guangzhou, Guangdong, China; 7 Department of Pathology, The Affiliated Guangdong Second Provincial General Hospital of Jinan University, Guangzhou, Guangdong, China

**Keywords:** Behçet’s disease, *GATA2* deficiency, HA20, *RELA* haploinsufficiency, systemic autoinflammatory diseases

## Abstract

**Objectives:**

The aim of this study was to characterize the genetic basis of Behçet’s disease (BD) and mimics in a pediatric cohort.

**Methods:**

We retrospectively studied 34 patients of pediatric BD and mimics who underwent whole-exome sequencing (WES) in our center from 2020 to 2025. Clinical characteristics and laboratory parameters of the patients with or without genetically defined systemic autoinflammatory diseases (SAIDs) were compared.

**Results:**

WES identified 10 probands with genetically defined SAID (the SAID+ group). Nine probands possessed pathogenic or likely pathogenic germline variants in *RELA*, *TNFAIP3* (encoding A20), or *GATA2*. One proband was found to have trisomy 8. Five of the SAID+ probands exhibited parentally inherited variants, while the remaining four probands possessed *de novo* variants. Compared to pediatric BD patients without genetically determined SAID (the ped-BD group, *n* = 24), the SAID+ group exhibited earlier disease onset and greater prevalence of recurrent fever episodes, intestinal involvement, and hematologic abnormalities. Laboratory studies revealed higher levels of inflammatory markers and serum cytokines including IL-1β, IL-2, IL-10, and TNFα in the SAID+ group. Corticosteroid treatment was commonly utilized in all patients, while the use of biologic DMARDs was more common in the SAID+ group.

**Conclusion:**

Pediatric patients with autoinflammatory diseases mimicking BD have heightened systemic inflammation and more severe organ involvement that necessitate intensified immunosuppressive regimens. Genetic evaluation should be considered for BD cases with early disease onset, recurrent fever, enteritis, intestinal ulcers, or hematologic abnormalities.

## Introduction

Behçet’s disease (BD) is a systemic inflammatory disease characterized by recurrent ulcerations and variable vasculitis that affects arteries and veins (1, 2). While susceptibility alleles have been identified, the cause of BD remains unknown. Most cases of BD occur in adults; juvenile patients with disease onset before 16 years of age account for approximately 2.7%–17.6% of reported cases (3). Although less common, features of BD have been described in monogenic diseases caused by pathogenic variants in *TNFAIP3*, *WDR1*, *ADA2*, *NCF1*, *AP1S3*, *GLA*, *TNFRSF1A*, *STAT1*, *ISG15*, and *ELF4* (4).

BD is often challenging to diagnose due to disease heterogeneity among patients and a wide spectrum of clinical manifestations that includes fever, oral and genital ulcers, skin rash, arthritis, uveitis, enteritis, and neurovascular involvement. With advances in immunology and genetics, a growing number of genetically defined systemic autoinflammatory diseases (SAIDs) have been discovered. These conditions are typically caused by genetic defects that result in spontaneous activation of the innate immune system. Interestingly, several monogenic SAIDs that involve aberrant activation of nuclear factor kappa beta (NF-κB) and production of tumor necrosis factor (TNF) are associated with features of BD. For example, haploinsufficiency of A20 (HA20) due to variants in *TNFAIP3* is a close mimic of BD with systemic inflammation and ulcerations throughout the GI tract (5). Compared with adult patients, intestinal ulcers were significantly more frequent in pediatric HA20 patients in a large Chinese cohort, which identified a total of 89 pathogenic *TNFAIP3* mutations (6). Patients that resemble BD have also been described for *RELA* haploinsufficiency and deficiency of adenosine deaminase 2 (DADA2) (7, 8).

Genetically defined SAIDs are characterized by strong familial heritability and early age of disease onset. Recent studies have shown that pediatric cases of BD may be explained by these conditions (2). In this study, we investigated the genetic basis of disease in a single center cohort of 34 pediatric patients with BD and compared the clinical manifestations and treatment response in patients with or without genetically defined SAIDs.

## Methods

### Study approval and patient enrollment

This study was approved by the Institutional Review Board of Guangdong Second Provincial General Hospital (Approval No. 2025-KY-KZ-006-01). Written informed consent was obtained from all participating families. A total of 51 pediatric patients with suspected or confirmed Behçet’s disease were seen at our center from 2020 to 2025. Among them, all 34 cases that had completed whole-exome sequencing were enrolled, and a retrospective analysis of their clinical data was performed. The remaining families declined participation due to the additional cost of genetic testing or for other reasons. On the basis of defining likely causal variants responsible for SAIDs, we divided the cohort into a SAID+ group (*n* = 10) and a ped-BD group (*n* = 24).

All patients have a Behçet-like phenotype, with recurrent oral ulcers and ≥2 of the additional manifestations (genital ulceration, skin inflammation, ocular inflammation, neurological features, vasculitis, gastrointestinal ulcers, arthritis, hemocyte abnormality, and recurrent fever). Two patients in the SAID group and all patients in the ped-BD group fulfilled the 2015 classification criteria for pediatric Behçet’s disease (9). Chart review was performed, and clinical characteristics, test results, treatment, and outcome measures were recorded. All patients had their laboratory data collected during the active phase of the disease.

Patients diagnosed with other forms of inflammatory arthritis and systemic autoimmune diseases or found to have positive autoantibodies including anti-extractable nuclear antigen antibodies, rheumatoid factor, and anti-cyclic citrullinated peptide antibodies were excluded from the study. Patients with the following conditions were also excluded from the study: systemic lupus erythematosus, Sjögren’s syndrome, anti-neutrophil cytoplasmic antibody-associated vasculitis, polyarteritis nodosa, pemphigus, and confirmed infection at the time of evaluation.

### Whole-exome sequencing

Genomic DNA used for whole-exome sequencing (WES) was extracted from peripheral blood using the Promega DNA Extraction Kit (AS1520). DNA (1 μg) was used for WES on Illumina NovaSeq platform with 100× coverage. Sequencing reads are mapped to the human reference genome (GRCh38) using the bwa (v0.7.17) and samtools (v1.2) software. Variants were called using the Genomic analysis toolkit (GATK) and were annotated by ANNOVAR. Variants with allele frequency >1% in public databases were filtered. Variants meeting pathogenic criteria of the American College of Medical Genetics (ACMG) were selected. Pathogenic variants in this study were confirmed by Sanger sequencing. The trisomy 8 variant was identified by increased coverage of chromosome 8. There was no copy number variation identified in other patients.

### Cytokine analysis

The levels of IL-1β, IL-2, IL-6, IL-8, IL-10, IL-17, interferon-γ, and TNF-α in plasma were analyzed and quantified using cytometric bead array (CBA) as per the manufacturer’s (BriCyte E6, Mindray, China) instructions.

### Statistical analysis

Statistical analyses were performed with GraphPad Prism 8.2.1. Quantitative variables were presented as mean ± standard deviation (SD). Comparisons of the differences of continuous variables were performed using the Mann–Whitney *U* test. A *p*-value <0.05 was accepted as statistically significant.

## Results

### Identification of genetic causes of pediatric BD

**Figure 1:**
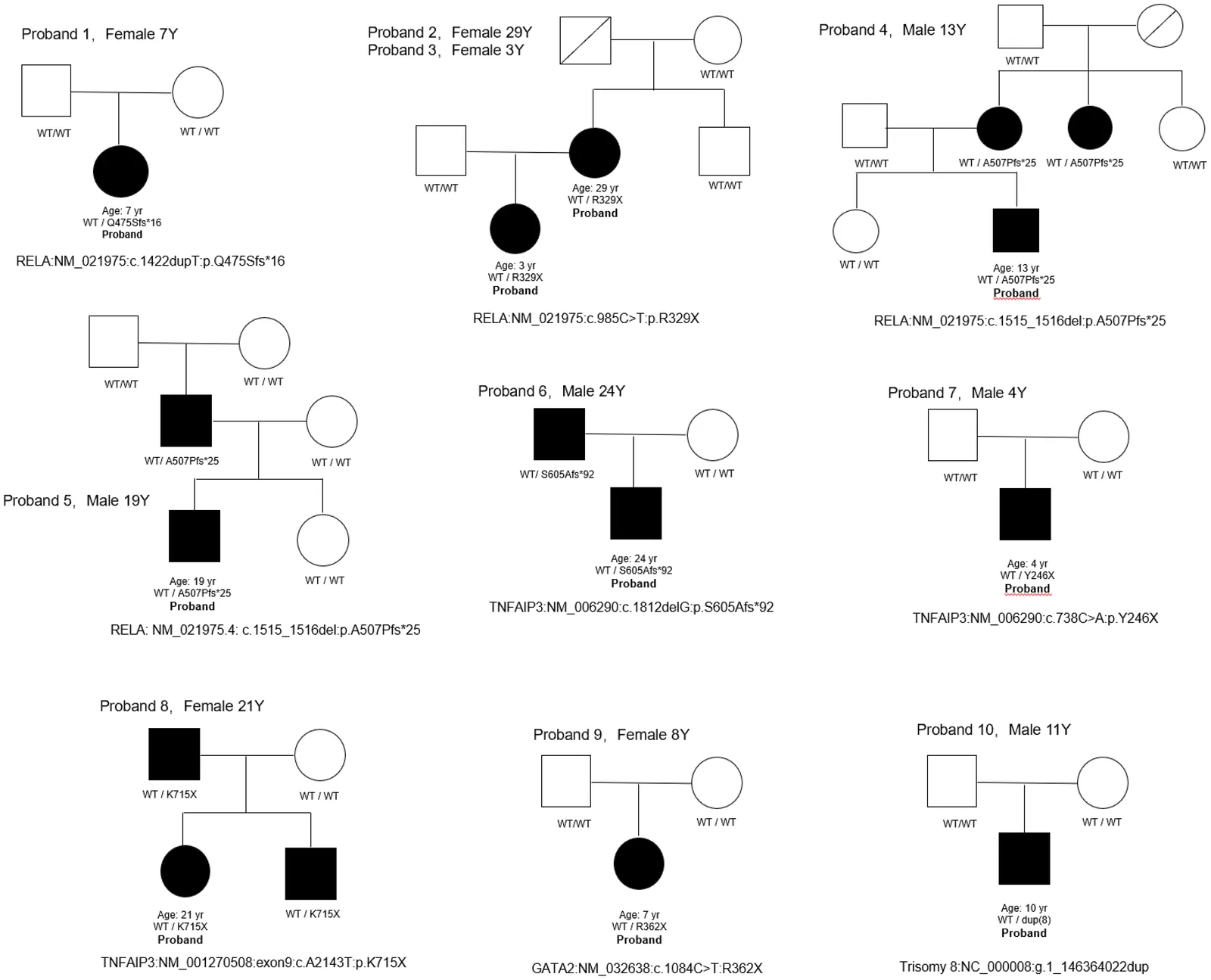
Pedigrees and details of the variants in the SAID group.

Here, we sought to identify the genetic basis of BD in 34 pediatric patients followed in our center. We analyzed WES for all patients and identified 10 proband with genetic findings associated with SAID. The probands’ clinical characteristics and treatment details are shown in **Tables 1**, **2**. Pedigrees and details of the variants from all nine kindreds are displayed in **Figure 1**. Nine proband possessed pathogenic or likely pathogenic germline variants in *RELA*, *TNFAIP3* (encoding A20), and *GATA2*, compatible with the diagnoses of *RELA* haploinsufficiency (five cases), haploinsufficiency of *A20* (HA20; three cases), and *GATA2* deficiency (one case), respectively. One proband was found to have trisomy 8. Five of the 10 probands exhibited parentally inherited variants (three cases with *RELA* haploinsufficiency and two cases with HA20), while the other 4 probands (P1, P7, P9, P10) possessed *de novo* variants and proband 2 was no applicable.

**Table 1:** Clinical characteristics of the SAID+ group.

Cases	Gender	Age at onset (years)	Age at diagnosis (years)	Gene mutation	Initial symptoms	Symptoms
P1	Female	5	6	*RELA* (*de novo*)	Oral ulcer	Recurrent oral aphthosis, genital ulcers, neutropenia
P2	Female	2	28	*RELA* (NA)	Oral ulcer	Recurrent oral aphthosis, fever, intestinal ulcer with perforation, arthritis
P3 (P2’s daughter)	Female	0.08	2.5	*RELA* (mother)	Oral ulcer	Recurrent oral aphthosis, cutaneous signs, intestinal ulcer, neutropenia, fever, arthritis, superficial fasciitis
P4	Male	0.75	12	*RELA* (mother)	Oral ulcer	Recurrent oral aphthosis, ocular signs, cutaneous signs, intestinal ulcer, fever
P5	Male	2	18	*RELA* (father)	Oral ulcer	Fever, recurrent oral aphthosis, genital ulcers
P6	Male	10	23	*TNFAIP3* (father)	Oral ulcer	Recurrent oral aphthosis, intestinal ulcer, fever
P7	Male	3	4	*TNFAIP3* (*de novo*)	Fever	Recurrent oral aphthosis, intestinal ulcer, fever
P8	Female	15	21	*TNFAIP3* (father)	Oral ulcer	Recurrent oral aphthosis, genital ulcers, gastric ulcer
P9	Female	4	6	*GATA2* (*de novo*)	Oral ulcer	Recurrent oral aphthosis, genital ulcers, intestinal ulcer, neutropenia, thrombocytopenia
P10	Male	2	8	Trisomy 8 (*de novo*)	Thrombocytopenia	Recurrent oral aphthosis, cutaneous signs, intestinal ulcer, thrombocytopenia, neutropenia, fever

NA, data not available.

**Table 2:** Treatment and prognosis of the SAID+ group.

Cases	Treatment before genetic diagnosis	Treatment post-genetic diagnosis	Treatment at the most recent follow-up	Follow-up (months)	Survival
P1	Colchicine, thalidomide	Colchicine, thalidomide, etanercept	Colchicine, etanercept	33	Yes
P2	Steroids, AZA, thalidomide, adalimumab	Steroids, tofacitinib, adalimumab, enteral nutrition solution	SMZ, upadacitinib	23	Yes
P3 (P2’s daughter)	MTX, etanercept	Steroids, MTX, adalimumab, infliximab, tocilizumab	Steroids, MTX, adalimumab, tofacitinib	36	Yes
P4	Steroids, colchicine, thalidomide	Steroids, colchicine, thalidomide, adalimumab	Colchicine	45	Yes
P5	HCQ, thalidomide	Steroids, HCQ, colchicine, thalidomide, apremilast	Apremilast	12	Yes
P6	Steroids, thalidomide, adalimumab	Thalidomide, SMZ adalimumab	Drug-free for 7 months	33	Yes
P7	Steroids, infliximab	Steroids, infliximab	Thalidomide	35	Yes
P8	Steroids, thalidomide, MTX, adalimumab	Steroids, thalidomide, MTX, adalimumab	Colchicine, MTX	12	Yes
P9	Steroids, thalidomide	Colchicine, thalidomide, etanercept	Drug-free after HSCT for 1 year	65	Yes
P10	Steroids, SMZ, adalimumab	Steroids, SMZ, CTX, infliximab	Steroids, SMZ, infliximab	32	Yes

MTX, methotrexate; HCQ, hydroxychloroquine; SMZ, sulfasalazine; AZA, azathioprine; CTX, cyclophosphamide; HSCT, hematopoietic stem cell transplantation.

### Clinical and laboratory features

We compared the clinical features of pediatric BD with or without genetically determined SAID (SAID+ and ped-BD groups, respectively). The clinical characteristics of both groups are summarized in **Table 3**. SAID+ patients showed earlier disease onset compared with their counterparts without SAID (4.4 ± 4.7 vs. 7.6 ± 4.4 years; *p* = 0.037). The male-to-female ratio was comparable between the two groups. Oral ulceration affected all patients and was the initial symptom in the majority of cases in both groups. Other initial presenting symptoms included fever (*n* = 2) and thrombocytopenia (*n* = 1) in the SAID+ group and genital ulcers (*n* = 2) and ophthalmia (*n* = 1) in the ped-BD group. Other common manifestations of the SAID+ group included intestinal involvement (8/10 vs. 8/24, *p* = 0.023), hematological abnormalities including neutropenia and thrombocytopenia (4/10 vs. 0/24, *p* = 0.005), and recurrent fever episodes (7/10 vs. 2/24, *p* = 0.001).

**Table 3:** Comparison of the clinical features of the SAID+ and ped-BD groups.

Clinical features	SAID+ (*n* = 10)	ped-BD (*n* = 24)	*P*
Sex (M/F)	5/5	14/10	0.718
Age onset (years)	4.38 ± 4.66	7.58 ± 4.35	0.037
Age of diagnosis (years)	12.75 ± 9.11	12.39 ± 6.05	0.889
Recurrent oral aphthosis (*n*)	10	24	>0.999
Genital ulcers (*n*)	4	18	0.112
Ocular manifestations (*n*)	1	9	0.215
Cutaneous manifestations (*n*)	4	16	0.252
Gastrointestinal manifestations (*n*)	8	8	0.023
Joint manifestations (*n*)	2	7	0.692
Neurological manifestations (*n*)	0	0	>0.999
Vascular signs (*n*)	0	4	0.296
Hematological abnormalities (*n*)	4	0	0.005
Fever (*n*)	7	2	0.001
Positive pathergy test (*n*)	0	1	>0.999
Lab			
C-reactive protein (mg/L)	22.4 (9.4, 73.7)	4.0 (1.2, 11.5)	0.005
Erythrocyte sedimentation rate (mm/h)	49.5 (29.3, 78.0)	19.0 (12.0, 41.0)	0.015
IL-1β (pg/mL)	3.7 (1.7, 12.0)	1.2 (0.20, 3.5)	0.029
IL-2 (pg/mL)	2.3 (1.7, 6.0)	0.8 (0.6, 2.0)	0.015
IL-6 (pg/mL)	4.4 (2.8, 8.7)	2.7 (1.4, 6.2)	0.103
IL-8 (pg/mL)	26.5 (5.9, 74.4)	6.1 (1.5, 21.6)	0.094
IL-10 (pg/mL)	3.5 (1.6, 7.6)	1.0 (0.8, 1.7)	0.002
IL-17 (pg/mL)	3.2 (1.6, 4.4)	1.6 (0.7, 2.8)	0.154
Tumor necrosis factor-α (pg/mL)	3.3 (2.9, 7.1)	2.0 (1.0, 4.0)	0.045
Interferon-γ (pg/mL)	3.1 (2.1, 9.8)	4.0 (1.1, 6.4)	0.751
ANA positive	7	7	0.054
Treatment			
Steroids (*n*)	8	18	>0.999
Colchicine (*n*)	4	12	0.714
Thalidomide (*n*)	7	19	0.666
csDMARDs (*n*)	4	11	>0.999
bioDMARDs (*n*)	9	9	0.008
JAKi (*n*)	2	3	0.618

IL, interleukin; csDMARDs, conventional synthetic disease-modifying antirheumatic drugs; bioDMARDs, biologic disease-modifying antirheumatic drugs; JAKi, Janus kinase inhibitor.

**Figure 2:**
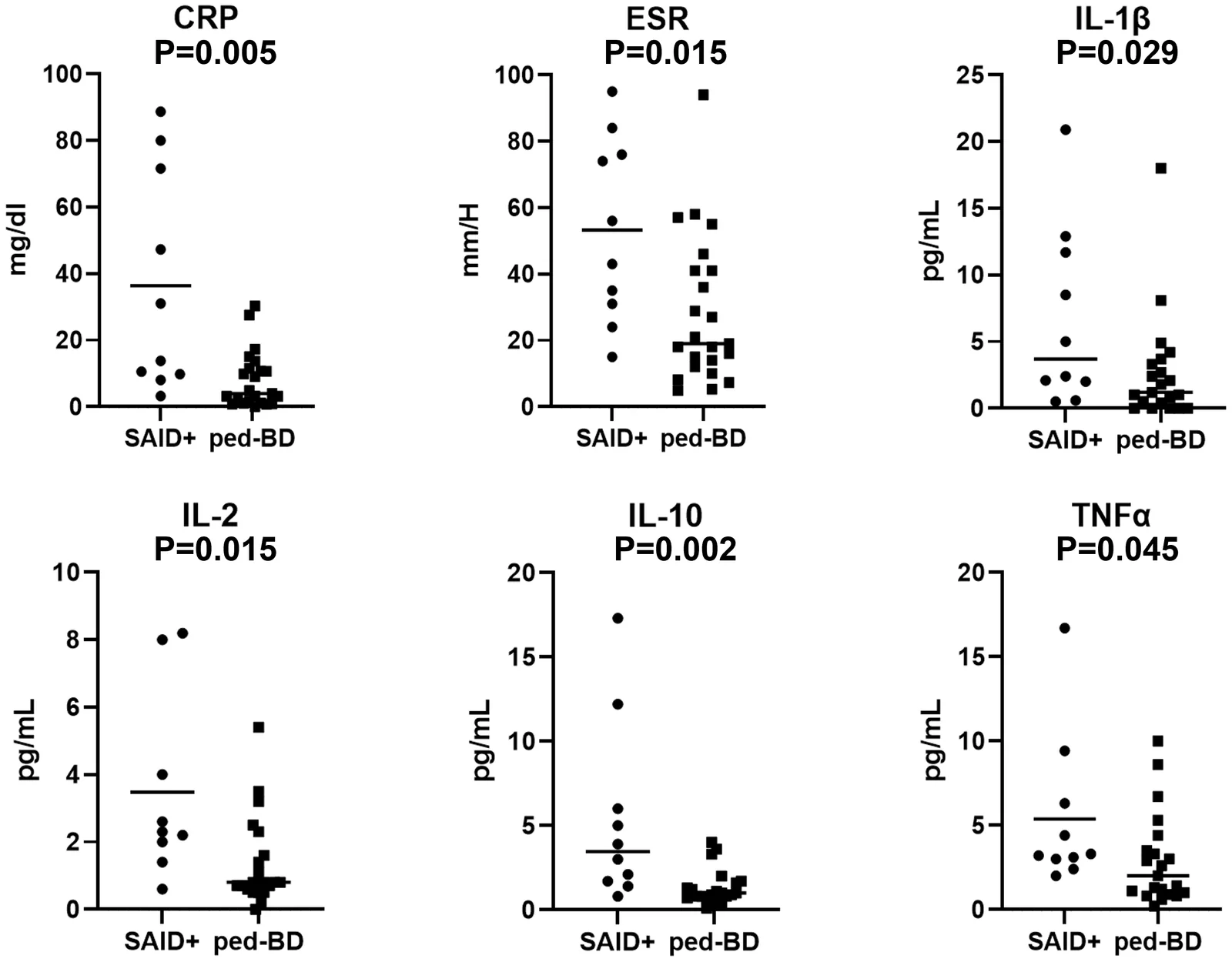
Compared laboratory indicators and cytokines with SAID+ and ped-BD.

Comparison of laboratory features (**Figure 2**) revealed higher levels of C-reactive protein [22.4 (interquartile range 9.4–73.7) vs. 4.0 (1.2–11.5) mg/L, *p* = 0.005] and erythrocyte sedimentation rate [49.5 (29.3–78.0) mm/h vs. 19.0 (12.0–41.0) mm/h, *p* = 0.015] in the SAID+ group compared with the ped-BD group. In line with these findings, SAID+ patients also exhibited higher plasma levels of IL-1β [3.7 (1.7–12.0) vs. 1.2 (0.20–3.5) pg/mL, *p* = 0.029], IL-2 [2.3 (1.7–6.0) vs. 0.8 (0.6–2.0) pg/mL, *p* = 0.015], IL-10 [3.5 (1.6, 7.6) vs. 1.0 (0.8, 1.7) pg/mL, *p* = 0.002], and tumor necrosis factor-α [3.3 (2.9, 7.1) vs. 2.0 (1.0, 4.0) pg/mL, *p* = 0.045]. The laboratory features of the SAID+ group were also displayed for each disease (**Supplementary Figure 1**). Taken together, these data showed that pediatric BD cases with SAID are associated with earlier disease onset and a trend toward more prominent systemic inflammation.

### Treatment and outcome

Medication usage in our cohort is summarized in **Tables 2**, **3**. Corticosteroids, colchicine, and thalidomide were the most used medications. Frequently used conventional synthetic disease-modifying antirheumatic drugs (csDMARDs) include methotrexate (MTX), sulfasalazine (SMZ), and hydroxychloroquine (HCQ). Most of the SAID+ patients were treated with biologic disease-modifying antirheumatic drugs (9/10 vs. 9/24, *p* = 0.008), including adalimumab (six cases), etanercept (three cases), infliximab (three cases), and tocilizumab (two cases). Janus kinase inhibitors (tofacitinib or upadacitinib) were given to five patients with refractory disease (two SAID+ patients and three ped-BD patients).

**Figure 3:**
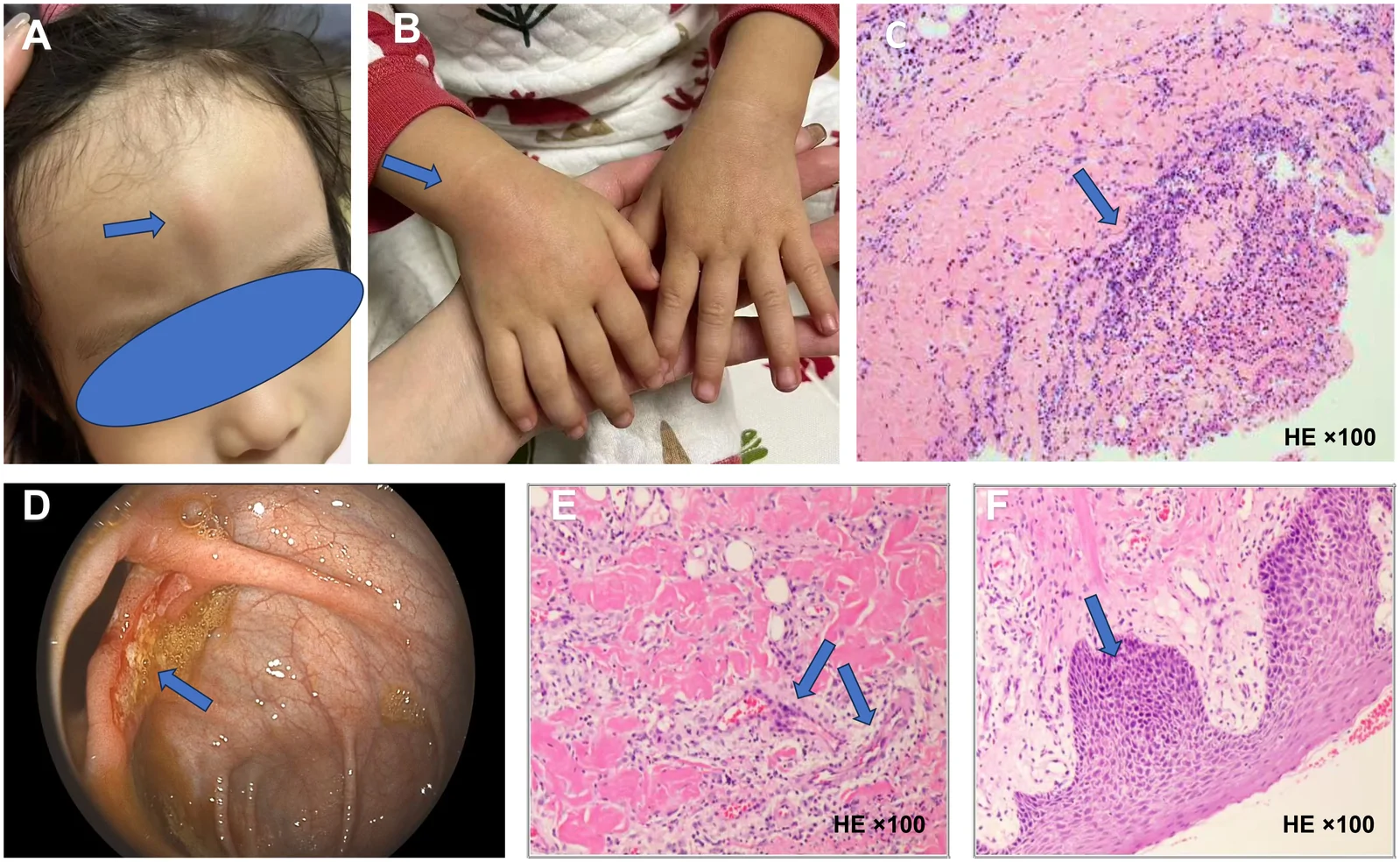
The clinical characteristics of proband 3 and proband 6 during inflammatory activity. Proband 3: **(A, B)** Multiple subcutaneous nodules of varying sizes with marked tenderness on palpation are palpable in the forehead, left submandibular region, posterior occipital area, right forearm, above the left trapezius muscle, and lumbodorsal region. **(C)** Pathological examination of the dorsal subcutaneous nodule reveals lymphocytic infiltration in the superficial fascia. Proband 6: **(D)** Colonoscopy revealed active ulceration in the mucosa of the ileocecal region. **(E, F)** Pathological examination of the oral ulcer showed perivascular infiltration of neutrophils and lymphocytes in the dermis, consistent with vasculitis, and focal acanthosis of the mucosal epithelium.

**Figure 4:**
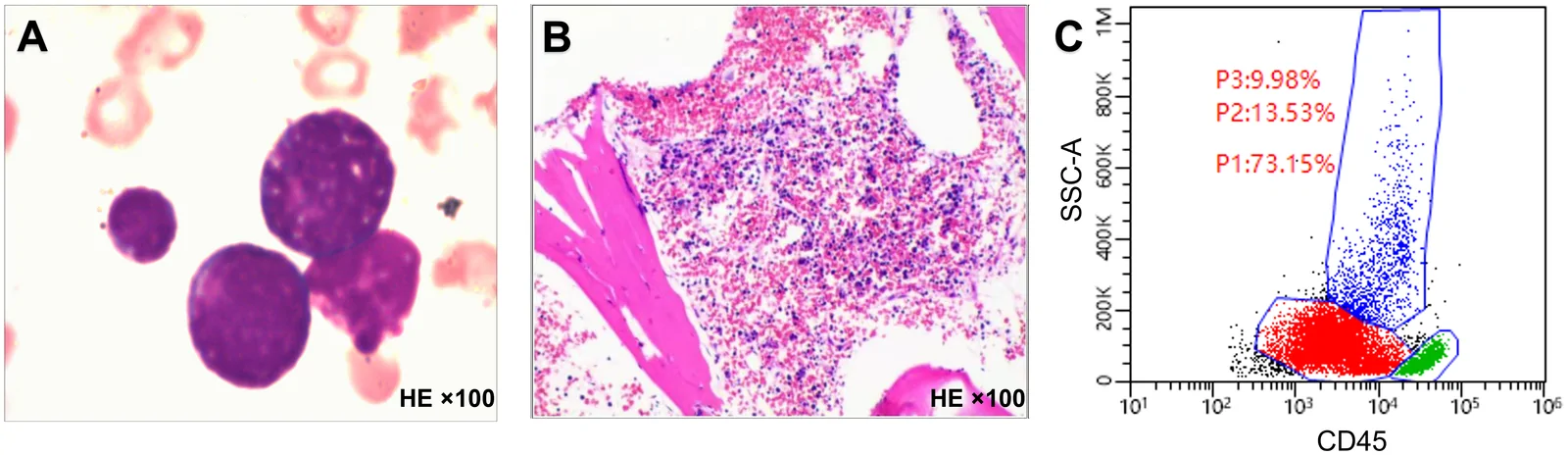
Proband 9 was diagnosed with acute lymphoblastic leukemia (ALL). **(A)** Bone marrow smear showed an increased proportion of the lymphoid lineage, with primitive and immature lymphoblasts accounting for approximately 63.0%. **(B)** Bone marrow histopathology revealed hypocellular hematopoiesis, with numerous primitive or immature lymphocytes in the medullary cavity. **(C)** Flow cytometry analysis indicated that P1 (red) represented primitive lymphocytes (73.15%), P2 (blue) represented mature granulocytes (13.53%), and P3 (green) represented mature lymphocytes (9.98%).

The 10 patients in the SAID+ group have been followed for an average of 32.6 ± 15.5 months, and outcomes are shown in **Table 2**. Eight patients responded well to treatment and remained in remission. One patient with *RELA* haploinsufficiency (proband 3) has experienced recurrent oral ulcers since birth, along with abdominal pain, diarrhea, fever, arthritis, and superficial fasciitis (**Figure 3**). Her disease was initially controlled with tocilizumab and corticosteroids. With the development of intestinal ulceration at 3 years of age, she was switched to infliximab and subsequently adalimumab with inadequate response. Marked improvement was seen after combining tofacitinib with adalimumab. The patient with *GATA2* deficiency (proband 9) was diagnosed with acute lymphoblastic leukemia at age 11 years (**Figure 4**) and underwent hematopoietic stem cell transplantation (HSCT). She has achieved 1 year of drug-free remission for features of BD since the transplantation. The patient with trisomy 8 (proband 10) is being considered for HSCT due to severe recurrent intestinal ulcers.

## Discussion

BD is a complex systemic inflammatory disease that typically develops during adulthood. A previous survey of BD in the Chinese population showed that children account for less than 5% of cases (10). Patients with pediatric BD often have a strong family history, suggesting a prominent role of genetics in driving early disease onset. In this study, we performed whole-exome sequencing on 34 patients of pediatric BD and found 9 patients with likely causal variants linked to monogenic SAIDs and 1 case with trisomy 8. Highlighting the importance of defining the genetic basis of pediatric BD, this subset of patients showed earlier disease onset, more severe clinical features, and a trend toward higher levels of systemic inflammation, all highlighting the need for more aggressive immunosuppressive therapy.

Since the initial description of familial Mediterranean fever, more than 50 monogenic SAIDs have been described (11). These conditions are characterized by dysregulated activation of innate immune pathways, leading to aberrant production of pro-inflammatory cytokines such as IL-1β, TNF, IL-6, and interferons. Interestingly, features of BD have been described in an expanding number of SAIDs linked to aberrant activation of NF-κB (10, 12). Zhou et al. (13) first linked pathogenic variants in *TNFAIP3* (encoding A20) to a BD-like inflammatory syndrome called HA20 in six unrelated families. A20 is a deubiquitinase that serves as a critical negative regulator of NF-κB signaling. Badran and Dedeoglu et al. described haploinsufficiency of *RELA* as another cause of familial BD. *RELA* encodes the NF-κB p65 subunit and forms a heterodimer with p50 to direct transcriptional activity. Haploinsufficiency of *RELA* leads to an imbalance of NF-κB signaling that augments TNF-driven mucosal apoptosis and impairs epithelial recovery (14). As a general theme of monogenic SAIDs, the clinical spectrum for HA20 and *RELA* haploinsufficiency is broad and also encompasses features of autoimmunity and immunodeficiency (15).

In addition to monogenic SAIDs, we also identified patients with trisomy 8 and *GATA2* deficiency. Trisomy of chromosome 8 is associated with systemic inflammation, autoimmunity, and increased risk for myeloid leukemia and myelodysplastic syndrome (MDS). BD with prominent gastrointestinal involvement is described in patients with trisomy 8, possibly related to the gene dosage of NF-κB regulators such as IKKβ in the duplicated chromosome (16). *GATA2* deficiency causes bone marrow failure, myelodysplastic syndrome, autoinflammation, and immunodeficiency (17). Our patient initially presented with BD at age 4 and developed ALL at age 11 years. Her features of BD fully resolved after HSCT.

We acknowledge that despite the overlapping clinical features and laboratory trends within the SAID+ group, the biology of each SAID is distinct and that grouping them together overlooks the differences among these complex conditions. On the other hand, the current sample size is not sufficient to further analyze these conditions individually. Focused cohort studies for each SAID are needed to understand the prevalence of BD features and whether BD is a common or rare disease phenotype.

Our findings of diverse genetic causes among pediatric BD patients are in agreement with a study from the United Kingdom (15). An algorithm for genetic evaluation of BD that includes assessment of both monogenic causes and the HLA risk alleles has been suggested (18). WES has the advantages of reduced cost, simplified workflow, and the potential to identify novel diseases (19). Despite the high concordance between WES and whole-genome sequencing (WGS), WES still has limitations. The coverage depth of WES is uneven to some extent across target regions, leading to a lower success rate of variant detection in these regions (20).

The main limitation of our study is the relatively small sample size from a single tertiary center. The proportion of patients with SAIDs is likely lower in the non-tertiary setting. Multicenter studies are needed to assess the prevalence of SAIDs among pediatric BD cases. The proportion and characteristics of the comparison groups may be affected by the one-third of pediatric BD cases in our center during the study period that did not undergo WES. HLA-B51 was not examined, and therefore, we are not able to address the contribution of this risk allele in our cohort. Lastly, we did not pursue functional studies for the SAID-associated variants. All of the variants (except for trisomy 8) are predicted to result in frameshift or truncation; loss-of-function variant in one allele is compatible with the described dominant mode of inheritance for HA20, *RELA* haploinsufficiency, and *GATA2* deficiency.

Currently, there are no consensus guidelines for the treatment of BD in children. Corticosteroids and csDMARDs remain the mainstay of treatment based on the experience from treating adults. Our experience shows that patients with BD secondary to SAID are more resistant to these conventional therapies and nine patients in the group required biologic DMARDs. Confirming a pathological role of NF-κB signaling and TNF production, early studies demonstrated that TNF antagonists are effective for individuals with HA20 and *RELA* haploinsufficiency. Interleukin-1 antagonists and JAK inhibitors have also shown benefits for patients with HA20 (21). A20 directly targets NF-κB signaling and the NLRP3 inflammasome. Although A20 is also described to regulate interferon signaling, the underlying mechanisms are incompletely characterized (22). Given the rarity of these conditions and heterogeneity among patients, an empiric approach to treatment is often used because the clinical response to a particular biologic DMARD is difficult to predict. One patient in our cohort with *RELA* haploinsufficiency failed tocilizumab and multiple TNF inhibitors before remission was attained using a combination of tofacitinib and adalimumab. Treatment options are also dictated by the variable availability of medications. IL-1 antagonists are commonly used in autoinflammatory diseases (11). Although they display an excellent long-term effectiveness and a favorable safety profile (23), the IL-1 antagonists anakinra and canakinumab are not yet available for clinical use for pediatric indications in China.

## Conclusions

Systemic autoinflammatory diseases can mimic BD, particularly when presenting early in life. The present study seeks to enhance the recognition of autoinflammatory diseases with features of BD among pediatricians. In young children with features of BD, genetic evaluation should be considered for the subset with early disease onset (the mean age of the SAID+ group was 4.4 years), recurrent fever, enteritis, intestinal ulcers, or hematologic abnormalities. Timely diagnosis and treatment will help minimize morbidity, as patients with SAIDs have more severe diseases and require more aggressive immunosuppressive therapy.

## Data Availability

The datasets presented in this study can be found in online repositories. The names of the repository/repositories and accession number(s) can be found in the article/**Supplementary Material**.
