# Correction: Genetically defined systemic autoinflammatory diseases in pediatric patients with Behçet’s disease

**DOI:** 10.3389/fimmu.2026.2007295

**Published:** 2026-10-12

**Authors:** Shaoling Zheng, Pui Y Lee, Qing Zhou, Xu Han, Xuechan Huang, Yukai Huang, Jiajun Chen, Chun Zheng, Xiaolin Fang, Jianhua Yu, Aiwu Wang, Xia Pan, Xiaomin Yu, Tianwang Li

**Affiliations:** 1 Department of Rheumatology and Immunology, The Affiliated Guangdong Second Provincial General Hospital of Jinan University, Guangzhou, Guangdong, China; 2 Division of Immunology, Boston Children’s Hospital, Harvard Medical School, Boston, MA, United States; 3 State Key Laboratory of Common Mechanism Research for Major Diseases, Institute of Basic Medical Sciences & School of Basic Medicine, Chinese Academy of Medical Sciences & Peking Union Medical College, Beijing, China; 4 Liangzhu Laboratory, Zhejiang University, Hangzhou, Zhejiang, China; 5 Department of Laboratory Medicine, The Affiliated Guangdong Second Provincial General Hospital of Jinan University, Guangzhou, Guangdong, China; 6 Department of Hematology, The Affiliated Guangdong Second Provincial General Hospital of Jinan University, Guangzhou, Guangdong, China; 7 Department of Pathology, The Affiliated Guangdong Second Provincial General Hospital of Jinan University, Guangzhou, Guangdong, China

**Keywords:** Behçet’s disease, *GATA2* deficiency, HA20, *RELA* haploinsufficiency, systemic autoinflammatory diseases

There was a mistake in **Figure 1** as published. Proband 8 was incorrectly labeled as “male”. The corrected **Figure 1** appears below.

**Figure 1:**
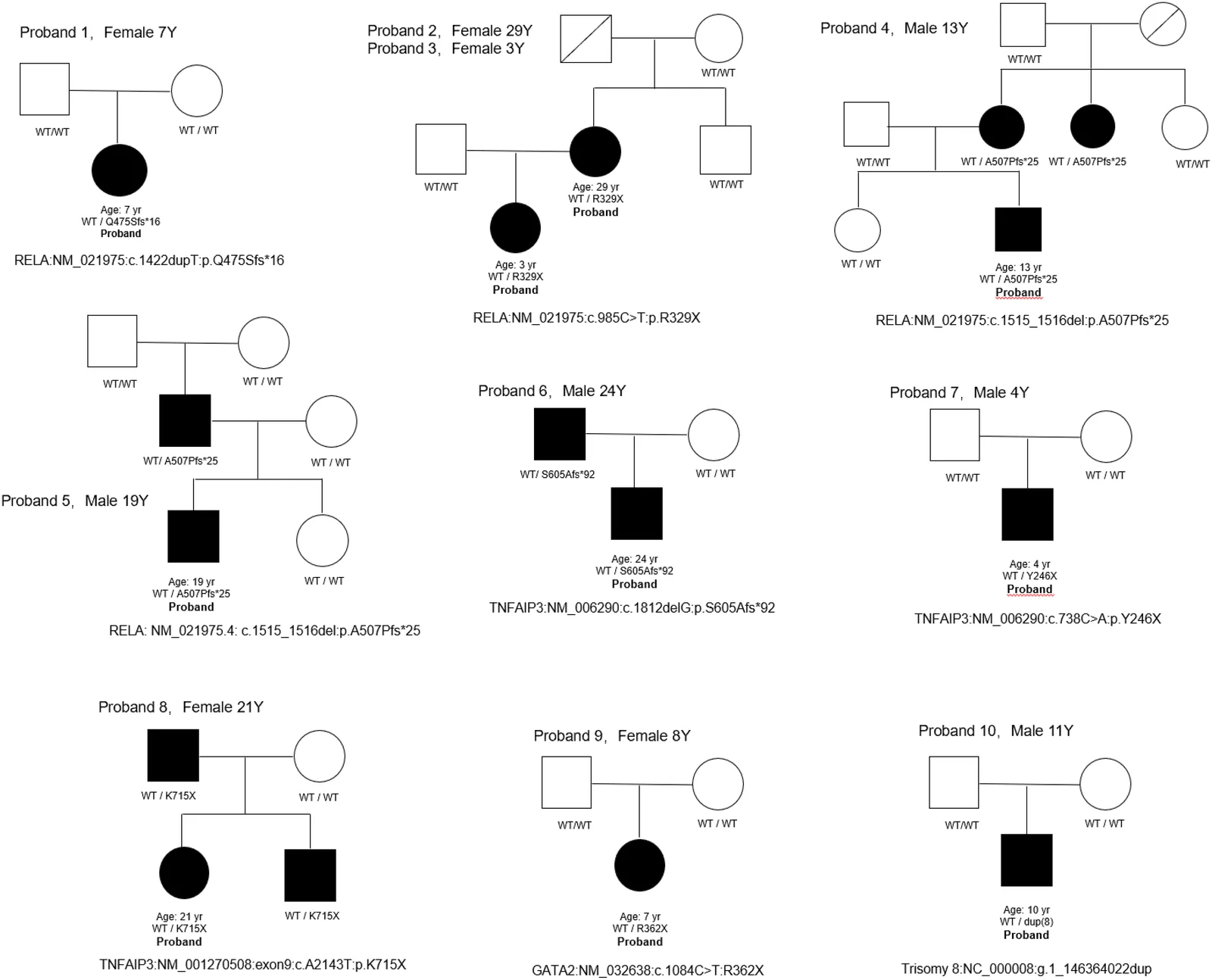
Pedigrees and details of the variants in the SAID group.

The original version of this article has been updated.

