# Supplementary material for: Genetically defined systemic autoinflammatory diseases in pediatric patients with Behçet’s disease
Source: Front Immunol. 2026 Aug 18;17:1897406. doi: 10.3389/fimmu.2026.1897406 (PMC13529686; doi:10.3389/fimmu.2026.1897406)
Supplement: Supplementary file 1 [file DataSheet1.pdf]

Supplemental Table 1 The laboratory features of the SAID+ group were further stratified according to each disease.

Lab	ped-BD<br>n=24	RELA<br>n=5	HA20<br>n=3	GATA2<br>n=1	Trisomy 8<br>n=1
C-reactive protein (mg/dL)	4.0(1.2, 11.5)	13.8(6.9, 59.9)	9.8(8.0, 71.6)	47.3	80.0
Erythrocyte Sedimentation Rate (mm/H)	19.0(12.0, 41.0)	35.0(27.5, 70.0)	74.0(15.0, 76.0)	95.0	43.0
IL-1β (pg/mL)	1.2(0.20, 3.5)	5.0(1.3, 10.1)	2.4(0.6, 20.9)	12.9	2.0
IL-2 (pg/mL)	0.8(0.6, 2.0)	3.1(1.0, 7.2)	2.6(1.4, 8.0)	2.3	2.0
IL-6 (pg/mL)	2.7(1.4, 6.2)	3.0(2.5, 16.0)	5.3(3.0, 7.6)	4.4	8.9
IL-8 (pg/mL)	6.1(1.5, 21.6)	26.5(11.1, 102.7)	6.1(1.5, 21.6)	28.4	2.0
IL-10 (pg/mL)	1.0(0.8, 1.7)	3.9(1.5, 5.5)	3.0(1,4, 12.2)	1.7	17.3
IL-17 (pg/mL)	1.6(0.7, 2.8)	2.3(0.3, 4.3)	3.1(2.0, 4.2)	3.2	9.0
Tumor necrosis factor-α (pg/mL)	2.0(1.0, 4.0)	6.3(2.6, 13.1)	3.3(2.4, 4.0)	3.2	3.0
Interferon-γ (pg/mL)	4.0(1.1, 6.4)	7.4(2.3, 12.8)	4.8(1.8, 7.1)	3.1	2.0

Supplemental Figure 1 The laboratory features of the SAID+ group were further stratified according to each disease.

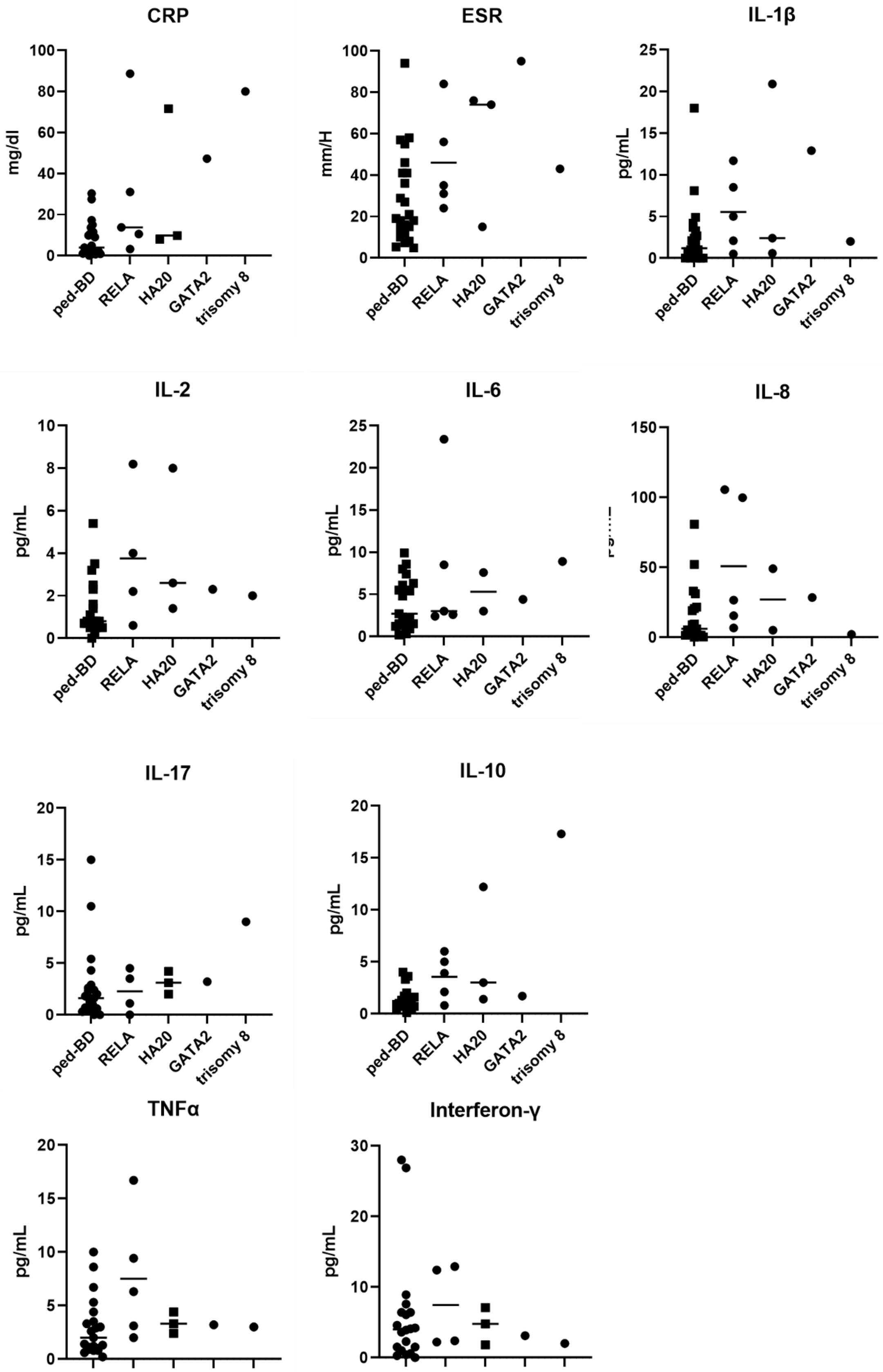

CRP
mg/dl
0
20
40
60
80
100
ped-BD
RELA
HA20
GATA2
trisomy 8
ESR
mm/H
0
20
40
60
80
100
ped-BD
RELA
HA20
GATA2
trisomy 8
IL-1β
pg/mL
0
5
10
15
20
25
ped-BD
RELA
HA20
GATA2
trisomy 8
IL-2
pg/mL
0
2
4
6
8
10
ped-BD
RELA
HA20
GATA2
trisomy 8
IL-6
pg/mL
0
5
10
15
20
25
ped-BD
RELA
HA20
GATA2
trisomy 8
IL-8
0
50
100
150
ped-BD
RELA
HA20
GATA2
trisomy 8
IL-17
pg/mL
0
5
10
15
20
ped-BD
RELA
HA20
GATA2
trisomy 8
IL-10
pg/mL
0
5
10
15
20
ped-BD
RELA
HA20
GATA2
trisomy 8
TNFα
pg/mL
0
5
10
15
20
ped-BD
RELA
HA20
GATA2
trisomy 8
Interferon-γ
pg/mL
0
10
20
30
ped-BD
RELA
HA20
GATA2
trisomy 8
